# Epidemiología y características clínicas de las mordeduras de serpientes venenosas en el norte de la Amazonía del Ecuador (2017-2021)

**DOI:** 10.7705/biomedica.6587

**Published:** 2023-03-30

**Authors:** Manuel Calvopiña, Elías Guamán-Charco, Karen Ramírez, Felipe Dávalos, Paola Chiliquinga, Sergio Villa-Soxo, René Oña-Vistin, Daniel Romero-Álvarez

**Affiliations:** 1 One Health Research Group, Facultad de Medicina, Universidad de las Américas, Quito, Ecuador Universidad de las Américas Universidad de las Américas Quito Ecuador; 2 Centro de Salud Tipo A “Santa Cecilia”, Ministerio de Salud Pública, Nueva Loja, Sucumbíos, Ecuador Ministerio de Salud Pública Nueva Loja Sucumbíos Ecuador; 3 Hospital General “Marco Vinicio Iza”, Ministerio de Salud Pública, Nueva Loja, Sucumbíos, Ecuador Ministerio de Salud Pública Nueva Loja Sucumbíos Ecuador; 4 Biodiversity Institute and Department of Ecology & Evolutionary Biology, University of Kansas, Lawrence, KS, USA University of Kansas University of Kansas Lawrence KS USA

**Keywords:** mordeduras de serpientes/epidemiología, animales venenosos, ecosistema Amazónico, Ecuador, Snake bites/epidemiology, animals, poisonous, Amazonian ecosystem, Ecuador

## Abstract

**Introducción.:**

En Ecuador, las mordeduras de serpientes venenosas son un problema de salud pública. Sin embargo, no existe información hospitalaria reciente desde la Amazonía.

**Objetivo.:**

Analizar retrospectivamente las características clínico-epidemiológicas de las mordeduras de serpientes en pacientes ingresados en un hospital de la Amazonía del Ecuador.

**Materiales y métodos.:**

Se llevó a cabo un estudio transversal en el hospital provincial de Nueva Loja (Sucumbíos), que colinda con Colombia, 2017-2021. La información sobre las variables demográficas, epidemiológicas y clínicas, y la condición al egreso hospitalario, se obtuvieron de la ficha epidemiológica del Ministerio de Salud Pública.

**Resultados.:**

En cinco años se hospitalizaron 147 pacientes (29,4 por año), sin que se presentaran muertes. Corresponden a 26, 34, 32, 29 y 26 casos, en el 2017, 2018, 2019, 2020 y 2021, respectivamente. Según el sexo, los más afectados fueron los hombres (n=99; 67,3 %), según el grupo etario, los de 21 a 30 años (n=28; 19,0 %) y, según la raza, los de etnia mestiza (n=94; 63,9 %), estudiantes y agricultores.

La mediana de edad fue de 28 años (rango: 4 a 81). Hubo mayor prevalencia en abril, junio y septiembre. Todos los accidentes fueron causados por serpientes Viperidae. Veinte (13,6 %) casos fueron leves, 90 (61,2 %), moderados, y 37 (25,2 %), graves. La mordedura fue más frecuente en los pies (45 casos). El 53,1 % recibió suero antiofídico antes de la hospitalización y en el 19,8 % de los pacientes se colocó un torniquete. La mediana de tiempo de llegada al hospital fue de 5 horas (rango: 1-192), y lo más frecuente fue entre 2 y 3 horas (41 casos). No se encontraron diferencias estadísticamente significativas según la gravedad del caso.

**Conclusiones.:**

Se evidenció una gran prevalencia de mordeduras de serpientes en el norte de la región amazónica-Ecuador, con mayor incidencia en la estación lluviosa y todas causadas por Viperidae. Es importante resaltar la mortalidad nula. Las campañas informativas sobre prevención y primeros auxilios, como la desmotivación del uso de torniquetes, serían fundamentales para reducir los casos, especialmente, en los grupos vulnerables.

## Abstract (en)

**Introduction:**

In Ecuador, poisonous snakebites are a public health problem. However, there is no recent hospital information from the Amazon.

**Objective:**

To retrospectively analyse the clinical-epidemiological characteristics of snakebites in patients admitted to a hospital in the Ecuadorian Amazon.

**Materials and methods:**

This is a cross-sectional study conducted at the Nueva Loja-Sucumbíos Provincial Hospital, bordering Colombia (2017-2021). Demographic, epidemiological and clinical variables, and condition at hospital discharge, were obtained from the epidemiological file of the *Ministerio de Salud Pública*.

**Results:**

In 5 years, 147 patients (29.4 per year) were hospitalized with no mortality.

They corresponded to 26, 34, 32, 29 and 26 cases, in 2017, 2018, 2019, 2020 and 2021, respectively. Men with 99 (67.3%), aged 21-30 years with 28, mixed race with 94 cases, students, and farmers, were the most affected. The most affected (99; 67.3%) were men, people 21 to 30 years-old (28; 19.0%), mestizos (94; 63.9%), students, and farmers.

The median age was 28 (range: 4-81) years. Prevalence was higher in April, June, and September. All accidents were caused by Viperidae snakes. Twenty (13.6%) cases were mild, (61.2%) were moderate and 37 (25.2%) were severe. The feet with 45 were the most bitten. Pre-hospitalization anti-venom serum was received by 53.1% and tourniquet by 19.8% patients. Median hospital arrival time was 5 (range 1-192) hours, mostly between 2-3 hours with 41 cases. No statistically significant differences were found considering the severity.

**Conclusions:**

A high prevalence of snakebites was evidenced in the north of the Amazon in Ecuador, with a higher incidence in the rainy season and all by Viperidae species. It is important to highlight the null mortality. Information campaigns on prevention and first aid, such as discouraging the use of tourniquets, especially among vulnerable groups.

Las mordeduras de serpientes son un importante problema de salud pública en el mundo, incluido el Ecuador. La Organización Mundial de la Salud (OMS) les considera como una de las 20 enfermedades tropicales olvidadas, cuya incidencia es mayor en África, Asia y Latinoamérica. Las mayores incidencias se presentan en países y regiones donde los sistemas de salud son débiles, principalmente, en comunidades tropicales remotas, poco desarrolladas y políticamente marginadas ^1^^,^^2^.

Ecuador está ubicado al noroeste de Suramérica, bordeado por el océano Pacífico al oeste. Por su situación geográfica en la zona tórrida, es atravesado por la línea ecuatorial, el clima es subtropical y tropical, y las ecorregiones Costa y Amazonía presentan abundante biodiversidad ^3^.

Ecuador tiene gran diversidad de serpientes venenosas (~36 especies) y una de las más altas prevalencias de accidentes ofídicos en el continente americano, concentradas en áreas con altitudes menores de los 2.500 m.s.n.m. ^4^^-^^6^. Dos familias son de interés medico: Viperidae (víboras) con 17 especies y Elapidae (corales y marinas) con 18 especies. En la Amazonía ecuatoriana, las especies venenosas que predominan son *Bothrops atrox* y *B. bilineatus smaragdinus*, conocidas popularmente como “equis o pitalala” y “lorito machacui”, respectivamente ^6^^,^^7^. Las mordeduras por serpientes del género *Micrurus* “corales” son raras ^4^^,^^6^^,^^8^.

En el Ecuador, las mordeduras de serpientes son de reporte epidemiológico obligatorio en el Subsistema de Vigilancia Epidemiológica del Ministerio de Salud Pública (SIVE-ALERTA-MSP). Estas se reportan principalmente en las regiones tropicales de la Amazonía y la Costa; según su gravedad, pueden requerir hospitalización ^6^.

Según los datos oficiales obtenidos del Instituto Nacional de Censos y Estadísticas Vitales (INEC), entre los años 2014 y 2019, la tasa de mortalidad fue de 0,07 (rango: 0,03-0,10) por 100.000 habitantes ^9^^-^^11^. La región con la tasa más alta de incidencia es la Amazonía, con 55-78 casos por 100.000 habitantes (4,9). El SIVE-ALERTA-MSP, durante el periodo 2016-2020, registró 7.569 casos con un promedio anual de 1.514 casos. Para el 2020 se reportaron 1.438 casos, de los cuales 532 (37 correspondieron a la región amazónica ^12^.

La Amazonía del Ecuador tiene una altitud bajo los 750 m.s.n.m. y comprende 116.588,10 km^2^ (45,5 %) del territorio continental. La población que vive en esta región son 956.699 (5,5 %) del total de la población ecuatoriana, 54 % reside en las áreas rurales ^13^ y está dividida políticamente en 6 provincias, siendo Sucumbíos la norteña que colinda con la Amazonía de Colombia y Perú; tiene clima tropical húmedo durante todo el año.

En la Amazonía ecuatoriana residen poblaciones autoidentificadas como indígenas (achuar, andoa, cofán, kichwa, secoya, siona, shiwiar, shuar, sapara y waorani) que corresponden al 33,1 % del total de su población; el resto son colonos mestizos y afroecuatorianos ^14^. Más del 45 % de los waoraníes han experimentado, al menos, una mordedura de serpiente; el 95 % de sus hombres adultos han sido mordidos en una ocasión y, aproximadamente, la mitad han sido mordidos más de una vez ^15^. En la Amazonía se han identificado, al menos, tres comunidades rurales densamente pobladas con alto riesgo de mordedura de serpientes ^4^.

Según la gravedad de la sintomatología, el protocolo “Manejo clínico del envenenamiento por mordeduras de serpientes venenosas y picaduras de escorpiones” del Ministerio de Salud Pública ^6^ clasifica a los accidentes ofídicos en leves, moderados y graves; además, enlista los signos y síntomas como manifestaciones clínicas locales, sistémicas, complicaciones y secuelas.

Las serpientes de la familia Viperidae producen sintomatología hematotóxica y miotóxica, mientras que, con las Elapidae, la sintomatología es neurotóxica ^6^. A nivel nacional, para el periodo 2015 a 2017 con un total de 4.661 casos, el promedio de gravedad se registró en el 52,5 %, el 34,6 % y el 13 % para casos leves, moderados y graves, respectivamente ^6^. Para el año 2020, 232 (16,13 %) casos se consideraron graves ^12^. El 99 % de las mordeduras fueron causadas por serpientes Viperidae, y afectaron mayoritariamente al sexo masculino y los grupos etarios de niños y adultos jóvenes de 20 a 49 años. Las ocupaciones más afectadas incluyeron a los agricultores, los cazadores y los jornaleros ^6^^,^^16^^,^^17^.

Según el Protocolo del Ministerio de Salud Pública (2017), el suero antiofídico es el único medicamento biológico recomendado para su tratamiento. Este es polivalente para serpientes de la familia Viperidae y producido en Costa Rica; el número de frascos por administrar depende de la gravedad del cuadro clínico. El Ministerio de Salud Pública recomienda evitar prácticas inadecuadas, como torniquetes, hielo local, electricidad, uso de hidrocarburos y emplastos, calor local, incisiones en el sitio de la mordedura, succión, etc. Las medidas de primeros auxilios deben limitarse a la inmovilización de la extremidad y al traslado rápido del paciente al hospital ^6^. La mayoría de los grupos indígenas creen en la medicina tradicional a base de brebajes preparados con hidrocarburos, yerbas y plantas medicinales, ofrecidas por curanderos y chamanes ^5^^,^^10^^,^^18^.

En el presente trabajo, reportamos los resultados, recomendaciones y conclusiones del análisis de las variables demográficas, epidemiológicas y clínicas, registradas en las fichas epidemiológicas de 147 pacientes ingresados en el hospital provincial ubicado en la norteña provincia de Sucumbíos, en la Amazonía del Ecuador, durante el periodo de cinco años de 2017 a 2021.

## Materiales y métodos

### Sitio y población de estudio

**Figura 1.**
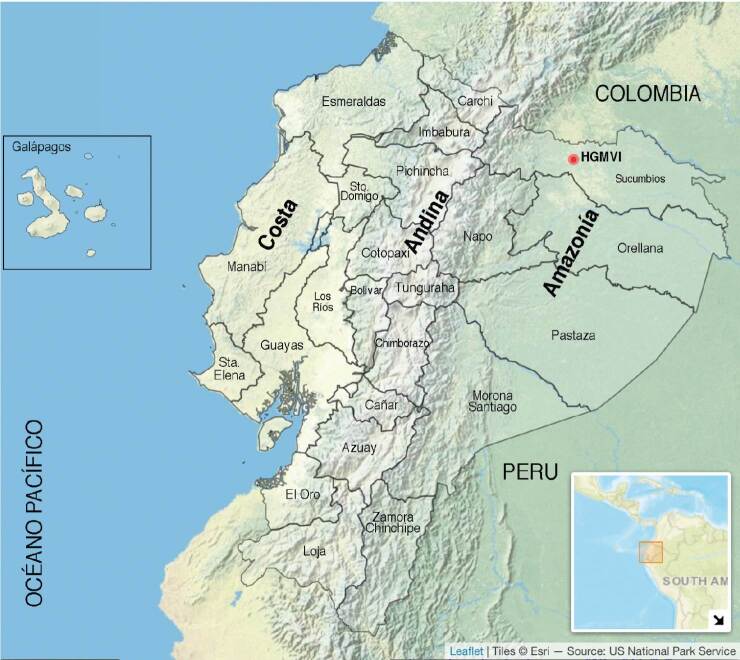
Mapa del Ecuador. Se diferencian las tres macrorregiones continentales: Costa, Andina y Amazonía, además de las islas Galápagos en el océano Pacífico. El mapa muestra las 24 provincias del país y la ubicación del Hospital General “Marco Vinicio Iza” (HGMVI, punto rojo) en la ciudad de Nueva Loja, provincia de Sucumbíos, limitando y continuando al norte y al este con la selva amazónica de Colombia y Perú, respectivamente.

El Hospital General “Marco Vinicio Iza” es un centro de salud público de nivel dos, perteneciente al Ministerio de Salud Pública del Ecuador, ubicado en la ciudad de Nueva Loja (latitud norte: 0.081861, longitud oeste: -76.886017) capital de la provincia de Sucumbíos, situado a 275 km de Quito. El hospital ofrece atención permanente y gratuita, y su cobertura incluye el noreste de la región amazónica del Ecuador (figura 1). Esta región se caracteriza por un clima lluvioso durante todo el año, más intenso de marzo a julio, seguidos de septiembre y octubre; los meses menos lluviosos son de diciembre a febrero ^19^.

Mapa elaborado con el paquete en Rstudio y Adobe Photoshop Elements.

Para este estudio, se incluyeron todos los pacientes que fueron hospitalizados con el Código Internacional de Enfermedades CIE-10 T63.0, que solo incluye mordeduras por serpientes venenosas ^20^. Los datos obtenidos y analizados corresponden al periodo de enero de 2017 a diciembre de 2021 (cinco años). Se consideraron pacientes de cualquier edad, sexo, etnia y nacionalidad, que se hospitalizaron con el código antes mencionado.

### Fuente de información

Los datos se obtuvieron retrospectivamente de la “Ficha clínico- epidemiológica por mordeduras de serpientes” mantenidas en el departamento de estadística del Hospital General “Marco Vinicio Iza”. La ficha clínico-epidemiológica es completada por el médico que recibe al paciente en emergencias y registra la fecha, los datos geográficos y demográficos, el tiempo de llegada al hospital, los primeros auxilios recibidos (succión, inmovilización, frío local, torniquete, incisiones, suero antiofídico o ninguno), el lugar del accidente y el de la residencia. Además, registra la especie de serpiente agresora: equis o pitalala (*Bothrops* spp., *Bothriopsis* o *Lachesis* spp.), lorito machacui (*B. bilineatus*) o coral (*Micrurus* spp.), el sitio anatómico de la mordedura, la gravedad, las manifestaciones clínicas locales, sistémicas y complicaciones, y la condición (vivo o muerto) al egreso hospitalario.

### Manifestaciones clínicas

Los síntomas y signos locales y sistémicos obtenidos de la ficha clínico- epidemiológica, permiten clasificar el accidente en tres grupos según su gravedad de acuerdo con los criterios del Ministerio de Salud Pública ^6^.

El primero, accidente leve, incluye edema de un segmento del miembro afectado, con un aumento menor de 4 cm en el diámetro del miembro afectado en comparación con el contralateral, con equimosis o sin ella, y sangrado escaso o nulo; el segundo, accidente moderado, incluye edema de dos a tres segmentos del miembro afectado, aumento del diámetro en el área afectada de más de 4 cm en comparación con el lado no afectado, equimosis, escasas flictenas y sangrado local; en el tercero, accidente grave, las mordeduras son en cabeza o cuello, el edema involucra más de 3 segmentos del miembro afectado, hay síndrome compartimental, áreas de necrosis local y flictenas, y las mordeduras causadas por *Lachesis* spp.

Las complicaciones y secuelas varían dependiendo de la gravedad, y comprenden celulitis, absceso en el sitio de mordedura, fasciotomía por síndrome compartimental, amputación, discapacidad física, deterioro neurológico o reacciones alérgicas. Además, se ha registrado hemorragia, lesión renal aguda, plaquetopenia, anemia, choque y coagulación intravascular diseminada. Las manifestaciones clínicas locales incluyen dolor, edema, equimosis, hematoma, flictenas, hemorragia por el sitio de la mordedura y necrosis; mientras que las manifestaciones sistémicas son: epistaxis, hematemesis, melena, gingivorragia, hematuria, mareos, síncope, sudoración, sialorrea, trismo, diplopía, hipotensión, oliguria, anuria, hipertensión arterial sistémica, náusea, vómito, taquicardia, fasciculaciones y visión borrosa ^6^.

### Análisis estadístico

Dos investigadores, de manera independiente, introdujeron los datos o variables en Excel 2013™. El análisis estadístico se realizó con el programa Jamovi ^21^. Se conformaron grupos etarios por décadas. Se estimó la frecuencia de cada variable y se utilizó estadística descriptiva para las variables cualitativas y cuantitativas. Se usó la prueba de ji al cuadrado para determinar si existe una diferencia según año de reporte, sexo, lugar de la mordedura o gravedad de la lesión, aceptándose un valor de p menor de 0,05 como estadísticamente significativo.

### Aspectos éticos

Esta investigación es parte del proyecto nacional “Estudio retrospectivo sociodemográfico, factores de riesgo, clínica y tratamiento de los accidentes ofídicos en el Ecuador, período 2017-2020”, aprobado por el Comité de Ética en Investigación con Humanos de SOLCA (Autorización N° 2.084.630). Se obtuvo la autorización escrita de la dirección del hospital. No se anotaron datos de identificación personal, sino que se utilizó un código numérico codificación numérica para cada paciente.

## Resultados

Durante los cinco años del estudio (2017-2021), se registraron 147 hospitalizaciones. De los 147, 99 (67,3 %) eran hombres y 48 (32,7 %) mujeres, con una mediana de 28 años de edad (rango: 4-81). El grupo de edad más afectado fue el de 21 a 30 años, con 28 (19 %) casos. Seis (4,1 %) pacientes eran de nacionalidad colombiana, y el resto, ecuatorianos. Ningún paciente falleció. Las dos ocupaciones más afectadas fueron los estudiantes, con 46 (31,3 %) casos, y los agricultores, con 43 (29,3 %) casos. Según los grupos étnicos, hubo 94 (63,9 %) mestizos, 50 (34 %) indígenas, 2 (1,4 %) afrodescendientes y 1 (0,7 %) mulato. La mediana de tiempo de llegada al hospital fue de 5 horas (rango: 1-192), y lo más frecuente fue entre 2 y 3 horas en 41 (27,9 %) casos (cuadro 1). Todos los pacientes provenían de zonas rurales del norte de la Amazonia.

**Cuadro 1:** Características demográficas de los 147 pacientes registrados con el código CIE-10 T63.0 en el Hospital General “Marco Vinicio Iza” (2017-2021)

Variables		n	(%)
Nacionalidad			
	Ecuatoriano	141	95,9
	Colombiano	6	4,1
Sexo			
	Masculino	99	67,3
	Femenino	48	32,7
Edad (años)			
	≤10	26	17,7
	11-20	26	17,7
	21-30	28	19,0
	31-40	22	15,0
	41-50	19	12,9
	51-60	13	8,8
	>60	13	8,8
Ocupación			
	Estudiante	46	31,3
	Agricultor	43	29,3
	Ama de casa	27	18,4
	Empleado	15	10,2
	Obrero	7	4,8
	Artesano	1	0,7
	Conductor	1	0,7
	Desempleado	1	0,7
	Profesor	1	0,7
	Ninguno	3	2,0
	Tercera edad	2	1,4
Grupo étnico			
	Mestizo	94	63,9
	Indígena	50	34,0
	Afrodescendiente	2	1,4
	Mulato	1	0,7
Tiempo de llegada al hospital (horas)			
	≤1	17	11,6
	2-3	41	27,9
	4-5	23	15,6
	6-11	34	23,1
	12-23	11	7,5
	≥24	21	14,3

En el cuadro 2, se registra el tipo de serpiente involucrada. La “Equis” de la familia fue identificada por 69 (46,9 %) pacientes, no hubo ninguna coral del género *Micrurus* y, en 66 (44,9 %) casos, no identificaron la serpiente. Veinte (13,6 %) casos fueron registrados como leves, 90 (61,2 %) como moderados y 37 (24,2 %) como graves.

**Cuadro 2:** Serpientes identificadas, características clínicas, complicaciones, secuelas y primeros auxilios recibidos por los pacientes hospitalizados por mordedura de serpiente venenosa en el Hospital General “Marco Vinicio Iza” (2017-2021)

Variables		n	(%)
Tipo de serpiente (N=121)			
	*Bothrops* spp. “equis o pitalala”	69	46,9
	Sin identificar	66	44,9
	*Bothriopsis* “lorito machacui”	11	7,5
	*Lachesis* "verrugosa"	1	0,7
Gravedad (N=147)			
	Leve	20	13,6
	Moderado	90	61,2
	Grave	37	25,2
Manifestaciones locales (N=147)			
	Dolor	142	96,6
	Edema	139	94,6
	Eritema	94	63,9
	Sangrado	27	18,4
	Equimosis	26	17,7
	Flictenas	12	8,2
	Necrosis	7	4,8
	Parestesias	7	4,8
Manifestaciones sistémicas (N=147)			
	Ninguno	110	74,8
	Náuseas	14	9,5
	Gingivorragia	11	7,5
	Vómito	11	7,5
	Mareo	10	6,8
	Hematemesis	4	2,7
	Sudoración	4	2,7
	Hipotensión	3	2,0
	Visión borrosa	3	2,0
	Hipertensión	2	1,4
	Epistaxis	1	0,7
	Hematuria	1	0,7
Complicaciones y secuelas (N=147)			
	Ninguna	88	59,9
	Anemia	21	17,4
	Absceso localizado	18	14,9
	Celulitis	14	11,6
	Reacción alérgica al suero antiofídico	10	8,3
	Síndrome compartimental	5	4,1
	Hemorragia	2	1,7
	Lesión renal aguda	2	1,7
	Plaquetopenia	5	4,1
	Choque	2	1,7
	Amputación	1	0,8
	Discapacidad física	1	0,8
	Deterioro neurológico	1	0,8
Primeros auxilios (N=147)			
	Suero antiofídico	78	53,1
	Ninguno	48	32,7
	Torniquete	29	19,7
	Succión	11	7,5
	Otros	9	6,1
	Inmovilización	6	4,1
	Incisión en sitio de mordedura	5	3,4
	Chamán o curandero	2	1,4

Las manifestaciones clínicas locales más comunes fueron dolor en 142 (96,6 %) casos, edema en 139 (94,6 %) y eritema en 94 (63,9 %). Por otro lado, 110 (74,8 %) casos no tuvieron manifestaciones sistémicas. Las manifestaciones sistémicas más frecuentes fueron náuseas en 14 (9,5 %) casos, gingivorragia en 11 (7,5 %), vómito en 11 (7,5 %) y mareo en 10 (6,8 %). En 88 (59,9 %) casos no se observó ninguna complicación; las más frecuentes fueron anemia en 21 (17,4 %) casos, seguida del absceso en el sitio de la mordedura en 18 (14,9 %) y celulitis en 14 (11,6 %). En relación con los primeros auxilios, la aplicación de suero antiofídico en 78 (53,1%) casos y el uso del torniquete en 29 (19,7 %), fueron las maniobras más usadas; en 48 (32,7 %) casos no se brindaron primeros auxilios.

En el cuadro 3, se puede observar que el mayor número de ampollas de suero antiofídico administradas fue de 16 para casos moderados y graves, y que el mayor número de casos, 34, se registraron en el 2018.

**Cuadro 3:** Análisis estadísticos entre gravedad (leve, moderado o grave), año de reporte, sexo y lugar anatómico de la mordedura. Porcentajes calculados con el total de cada fila. Las filas con valores de 0 fueron descartadas para el análisis (*). Las comparaciones entre leve *Vs*. moderado, leve Vs. grave y moderado *Vs*. grave, no mostraron resultados estadísticamente significativos (datos no presentados).

Variables		Leve (n, %)	Moderada (n, %)	Grave (n, %)	Total (n, %)	GL	X^2^	p
Año (N=147)								
	2017	5 (19,2)	13 (50)	8 (30,8)	26 (100)	4	8,16	0,23
	2018	7 (20,6)	22 (64,7)	5 (14,7)	34 (100)			
	2019	11 (34,4)	15 (46,9)	6 (18,8)	32 (100)			
	2020	10 (34,5)	17 (58,6)	2 (6,9)	29 (100)			
	*2021	0 (0)	18 (69,2)	8 (30,8)	26 (100)			
Sexo (N=147)								
	Femenino	8 (16,7)	30 (62,5)	10 (20,8)	48 (100)	2	1,04	0,59
	Masculino	12 (12,1)	60 (60,6)	27 (27,3)	99 (100)			
Lugar de la mordedura (N=147)								
	*Cabeza	0 (0)	0 (0)	3 (100)	3 (100)	6	4,51	0,61
	*Torax	1 (25)	3 (75)	0 (0)	4 (100)			
	Brazo	1 (33,3)	1 (33,3)	1 (33,3)	3 (100)			
	Antebrazo	0 (0)	3 (60)	2 (40)	5 (100)			
	Mano	5 (12,2)	29 (70,7)	7 (17,1)	41 (100)			
	*Muslo	0 (0)	4 (57,1)	3 (42,9)	7 (100)			
	Pierna	4 (10,3)	24 (61,5)	11 (28,2)	39 (100)			
	Pie	9 (20)	26 (57,8)	10 (22,2)	45 (100)			
Número de ampollas de suero		2 a 4	4 a 16	4 a 16	36 (100)			
antiofídicos aplicadas* (N=133)		(11,1)	(44,4)	(44,4)				

**Figura 2.**
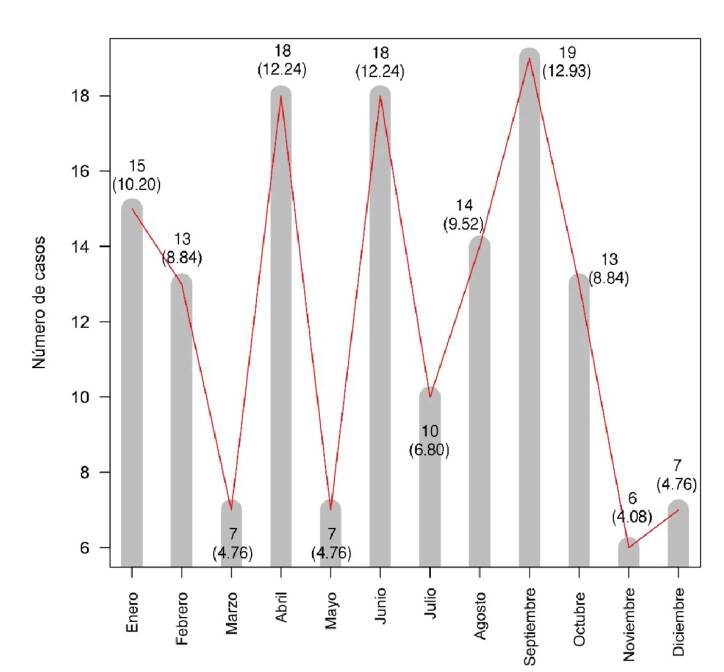
Frecuencia mensual de las 147 mordeduras de serpientes reportadas en el Hospital General “Marco Vinicio Iza” entre 2017 y 2021. Los números representan el número de casos por mes con el porcentaje entre paréntesis.

**Figura3.**
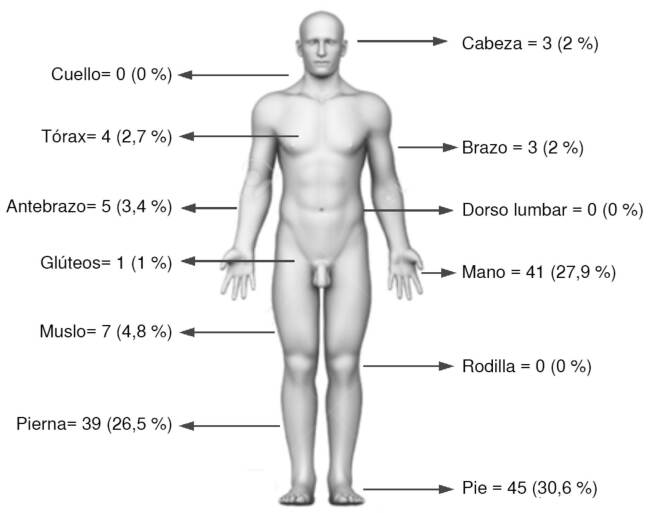
Distribución anatómica de las mordeduras de serpientes en los 147 pacientes ingresados en el Hospital General “Marco Vinicio Iza” (2017-2021)

Las mordeduras de serpientes ocurrieron con mayor frecuencia durante los meses de abril, con 18 (12,2 %) casos, de junio, con 18 (12,2 %), y de septiembre, con 19 (12,9 %) (figura 2). Según su localización, las mordeduras fueron más frecuentes en los pies, con 45 (30,6 %) registros, seguidos de las manos con 41 (27,9 %) y las piernas con 39 (26,5 %) (figura 3). No se encontraron diferencias estadísticamente significativas en ninguna de las variables analizadas (cuadro 3).

## Discusión

Las 29,4 hospitalizaciones anuales durante los últimos cinco años en el Hospital General “Marco Vinicio Iza”, demuestran que las mordeduras de serpientes en esta región del norte amazónico del Ecuador son endémicas y constituyen un problema de salud pública. Pocos y desactualizados son los estudios en la Amazonía ecuatoriana ^7^^-^^10^^,^^15^^,^^22^^,^^23^.

Es importante enfatizar que los casos analizados en este estudio son de pacientes sintomáticos hospitalizados por mordedura de serpientes venenosas. Los asintomáticos y la mayoría de los casos leves son manejados en los centros de salud de nivel uno que se encuentran distribuidos en las zonas rurales periféricas de la provincia de Sucumbíos. Los estudios a nivel nacional ubican a la región amazónica como la más afectada, con 55 a 78 casos por 100.000 habitantes ^9^. Siendo este un hospital ubicado cerca de la frontera con Colombia, se hospitalizaron 6 (4,1 %) ciudadanos colombianos.

El perfil epidemiológico de los hospitalizados en este estudio corresponde a hombres agricultores que viven en zonas rurales, económicamente activos (21 a 40 años), con la mayoría de las mordeduras en los miembros inferiores, perfil similar al reportado en otros estudios de la Amazonía ecuatoriana y en otros países de la región ^5^^,^^7^^,^^9^^,^^10^^,^^24^. Sin embargo, en este estudio, los estudiantes (46; 31,3 %) y las amas de casa (27; 18,4 %) siguieron en frecuencia a los agricultores, poblaciones no registradas como importantes en estudios previos. Potencialmente, esto se debe a las largas distancias que caminan los estudiantes por senderos en la selva para llegar a las unidades educativas. Además, en la cultura indígena de la Amazonía, las actividades de agricultura en el cuidado de la “chacra” (cultivos de yuca, plátano, maíz, etc.) se encomiendan a las mujeres.

Ambos grupos poblacionales deberán incluirse entre aquellos con factores de riesgo para mordedura de serpientes y deberían tomarse en cuenta para programas de educación y prevención. Asimismo, sugerimos que la actividad agrícola debería considerarse un factor de riesgo ocupacional para los accidentes ofídicos.

El 61,2 % de los pacientes hospitalizados fueron catalogados como casos moderados y, el 25 %, como graves. Los casos leves son tratados en los centros de salud de atención primaria ubicados en los cantones periféricos, que no tienen suficientes sueros antiofídicos para tratar casos moderados, graves o con complicaciones, por lo que son transferidos a este hospital provincial. Es por este motivo que los 13,6 % casos leves contrastan con los datos a nivel nacional, donde predominan con el 52,5 %, mientras que el número de casos graves reportados en este estudio (25,2 %) es superior al reportado a nivel nacional (13 %) ^6^^,^^25^.

Entre las discapacidades registradas en este estudio están la amputación, la discapacidad física y neurológica que se presentaron en un caso cada una, confirmando así que esta enfermedad tropical puede dejar secuelas permanentes ^26^. Además, las complicaciones más frecuentes fueron anemia, abscesos en el sitio de la mordedura y celulitis. Tanto las complicaciones como las secuelas pueden atribuirse al retraso en acudir a un centro de atención de referencia. Aquí, 21 (14,3 %) pacientes acudieron a las 24 y hasta las 96 horas después de la mordedura. Cabe mencionar que un manejo inadecuado de las mordeduras, realizado en casa por familiares y vecinos o por curanderos y chamanes debido a las creencias culturales, especialmente en el caso de la población indígena, podría también contribuir a la presentación de complicaciones y secuelas.

En el presente estudio, la mortalidad fue del 0 %, lo que coincide con la menor letalidad reportada a nivel nacional. Así, entre los años 2001 y 2007, el INEC notificó 61 muertes que representan una tasa de letalidad del 0,62 %, mientras que, entre 2014 y 2016, bajó a 0,058 muertes por 100.000 habitantes ^5^, aunque la región amazónica registró el mayor número de muertes por 100.000 habitantes ^9^. En estudios anteriores de los años 1991 y 2003 en hospitales de las provincias del sur de la Amazonía, corroboran que la tasa de mortalidad era alta, con el 5,4 % y el 2,9 %, respectivamente ^10^^,^^11^.

Consideramos que la mortalidad nula reportada aquí y baja a nivel nacional se debe a que el Ministerio de Salud Pública ha demostrado particular interés en la compra y provisión de suficiente número de sueros antiofídicos para los hospitales y centros de salud de atención primaria ubicados en las áreas rurales, y en la difusión del protocolo obligatorio de buenas prácticas de manejo de mordeduras de serpientes ^6^.

En este estudio, corroboramos que el 53,1 % de los pacientes llegaron a este hospital provincial con 4, 8 y 12 ampollas de suero antiofídico inyectadas como primeros auxilios recibidos en centros de salud periféricos. Además, la instauración de la cátedra de Medicina Tropical en la carrera de Medicina de algunas universidades y la buena práctica médica de los profesionales de la salud con experiencia en el manejo del accidente ofídico, han contribuido a que la atención de urgencia y el manejo hospitalario sean oportunos y eficaces, lo cual permite evitar muertes y complicaciones.

La mortalidad 0 registrada en el Hospital (2017-2021) se alinea con los objetivos planteados por la Organización Mundial de la Salud (OMS), específicamente, de reducir las muertes y la discapacidad por mordeduras de serpiente en un 50 % antes del 2030 ^2^. Sin embargo, algunos autores consideran que en la Amazonía sí hay muertes y que posiblemente hay subregistro de las mismas en los datos del Ministerio de Salud Pública ^10^^,^^16^^,^^22^. Una de las razones es la creencia en la medicina tradicional y en la “cura” con chamanes, especialmente, de los grupos indígenas que reemplazan la atención clínica y, por tanto, las muertes ocurren en la comunidad. Este fenómeno puede ser acrecentado por las grandes distancias para llegar a los centros de atención médica ^5^^,^^18^. En el presente estudio, solo 2 (1,4 %) pacientes acudieron al chamán o al curandero, ambos indígenas kichwas.

Las mordeduras se presentaron con mayor frecuencia en los meses de abril, junio y septiembre, precisamente los meses más lluviosos en esta región ^19^; esta tendencia se corrobora en otros estudios regionales ^10^. Creemos que esta tendencia se mantendrá o aumentará en el tiempo por las características ecológicas de la Amazonía: exuberante vegetación tropical, hábitat ideal para las serpientes venenosas, además de la migración humana hacia áreas selváticas en búsqueda de tierras para cultivo.

El mayor número de casos en la estación lluviosa se correlaciona con el periodo de reproducción de las serpientes y la mayor disponibilidad de presas, puesto que el incremento de agua en los ríos y pantanos determina que las serpientes y sus presas abandonen las madrigueras, incrementando así el encuentro con los humanos, quienes también incrementan las actividades agrícolas y cacería en estas épocas ^24^.

En concordancia con la distribución de las serpientes ^6^ y coincidiendo con los estudios anteriores realizados en la región amazónica tanto de Ecuador como de otros países ^24^, en este estudio, encontramos a la familia Viperidae, género *Bothrops* como la más frecuentemente implicada en los envenenamientos ofídicos. Así, *Bothrops* spp. fue identificada en el 46,9 %. Sin embargo, en el 44,9 % de los casos no se identificó la serpiente agresora, similar a lo evidenciado en el estudio de Sucua, al sur de la Amazonía ecuatoriana ^10^.

Un solo paciente identificó al género *Lachesis* spp. como causante de la mordedura. El envenenamiento por *Lachesis* spp. debe ser manejado y tratado como de máxima gravedad ^6^. No se reportó ningún caso por *Micrurus* (corales) de la familia Elapidae, lo cual concuerda la rareza de estos accidentes según datos del Ministerio de Salud Pública ^6^ y esto fue confirmado clínicamente sin sintomatología de neurotoxicidad. Sin embargo, existen reportes anecdóticos de mordeduras y la presencia de especies como *Micrurus helleri* en la región amazónica ^6^^,^^7^^,^^8^. Las especies de la familia Viperidae implicadas está acorde con las que predominan en la Amazonía ecuatoriana, *Bothrops atrox* y *B. bilineatus smaragdinus*^6^^,^^7^.

El predominio de mordeduras en los pies, con 45 (30,6 %) casos, y en las piernas, con 39 (26,5 %), reportado en este y otros estudios ^7^^,^^27^, hace notar la relevancia de la educación sobre la importancia de usar botas de caucho o zapatos altos durante las caminatas y actividades agrícolas, como medida preventiva. Sin embargo, las mordeduras en regiones más altas del cuerpo, como las manos (n=41; 27,9 %), podrían estar asociadas con la presencia de *B. bilineatus smaragdinus* que, por su color verde, se camuflan en las hojas de plantas como el café y que pueden agredir durante la cosecha.

Es importante notar que 81 (55,1 %) pacientes acudieron al hospital en las primeras cinco horas después de la mordedura, un tiempo corto, que puede deberse a la disponibilidad de ambulancias aéreas y marítimas, y a la mejor infraestructura terrestre y telefonía celular en la región, factores que también creemos que influyeron en la ausencia de mortalidad. Sin embargo, 21 (14,3 pacientes arribaron después de 24 horas; esta demora pudo presentarse en casos provenientes de las áreas selváticas más remotas y los referidos de centros de salud básicos ubicados en los cantones del interior de la selva, y son los dos indígenas que acudieron a los chamanes o curanderos. Por lo tanto, sugerimos incrementar la divulgación de información sobre mordeduras de serpientes en regiones rurales y, tal vez, en el lenguaje propio de los indígenas.

La falta de información y educación sobre mordeduras de serpientes en los pobladores de esta región se reflejó en este estudio: se usó el torniquete en 29 casos, y la incisión y succión en 5 de los 147 casos. Solo en 6 pacientes se empleó la inmovilización, una estrategia que si está recomendada en las guías del Ministerio de Salud Pública ^6^. Se debería informar que el uso del torniquete, los cortes o la succión, aumenta el riesgo de síndrome compartimental, isquemia, necrosis, hemorragia e infección, terminando en cirugías y amputaciones ^28^.

Recomendamos la difusión de medidas correctas y el acudir pronto a los centros de salud que sí disponen de sueros antiofídicos. Esto debería divulgarse por los medios de comunicación masivos, como la radio y televisión locales. Además, los médicos y enfermeras rurales deberían reforzar los conocimientos durante las consultas o en reuniones comunitarias.

Aunque no existen estudios controlados sobre su eficacia, el suero antiofídico recomendado y empleado en todo el Ecuador es el liofilizado, importado desde Costa Rica. Este lo distribuye gratuitamente el Ministerio de Salud Pública a todos los centros de salud y hospitales de áreas endémicas, es polivalente para las serpientes de la familia Viperidae, pero no es antídoto para las Elapidae (por ejemplo, las corales), que también están presentes en Ecuador, tanto en la Amazonía como en la Costa ^6^.

En 1995, en un estudio en la Amazonía ecuatoriana se determinó que el suero antiofídico del Laboratorio Butantan (Brasil), seguido del producido en Ecuador (Instituto Izquieta Pérez, Guayaquil), presentaban mayor eficacia ^23^. Infortunadamente, el Ecuador dejó de producir este suero desde hace 10 años. Recomendamos retomar la producción nacional con venenos de serpientes locales que, incluso, disminuiría los costos de importación y aseguraría tanto la oportuna disposición como el abastecimiento en todo el país ^29^. Se presentaron reacciones alérgicas a este suero antiofídico en 10 (8,3 %) pacientes, pero sin fallecimientos gracias al cumplimiento de los protocolos de manejo recomendadas por el Ministerio de Salud Pública ^6^ y la disposición oportuna de los fármacos.

En conclusión, los resultados del análisis de las 147 mordeduras de serpientes venenosas en el Hospital General “Marco Vinicio Iza” evidencian que el accidente ofídico es prevalente y de preocupación en esta región amazónica del Ecuador. Además, junto con los grupos de riesgo identificados (agricultores, amas de casa y jóvenes estudiantes) justifican la implementación de programas de educación en primeros auxilios a través de medios de comunicación masiva, y continuar con la provisión suficiente y oportuna de los sueros antiofídicos por las autoridades de salud, pero también, el potencial beneficio de producir sueros antiofídicos localmente.
